# Pre-existing antibodies directed against a tetramerizing domain enhance the immune response against artificially stabilized soluble tetrameric influenza neuraminidase

**DOI:** 10.1038/s41541-022-00435-7

**Published:** 2022-01-27

**Authors:** João Paulo Portela Catani, Emma R. Job, Tine Ysenbaert, Anouk Smet, Satyajit Ray, Lauren LaRue, Svetlana Stegalkina, Mario Barro, Thorsten U. Vogel, Xavier Saelens

**Affiliations:** 1 VIB-UGent Center for Medical Biotechnology, VIB, B-9052 Ghent, Belgium; 2 Department of Biochemistry and Microbiology, Ghent University, B-9000 Ghent, Belgium; 3 Sanofi Pasteur, Research North America, Cambridge, MA USA; 4 Present Address: Janssen Infectious Diseases and Vaccines, Janssen Research and Discovery, Beerse, Belgium

**Keywords:** Protein vaccines, Protein vaccines

## Abstract

The neuraminidase (NA) is an abundant antigen at the surface of influenza virions. Recent studies have highlighted the immune-protective potential of NA against influenza and defined anti-NA antibodies as an independent correlate of protection. Even though NA head domain changes at a slightly slower pace than hemagglutinin (HA), NA is still subject to antigenic drift, and therefore an NA-based influenza vaccine antigen may have to be updated regularly and thus repeatedly administered. NA is a tetrameric type II membrane protein, which readily dissociates into dimers and monomers when expressed in a soluble form. By using a tetramerizing zipper, such as the tetrabrachion (TB) from *Staphylothermus marinus*, it is possible to stabilize soluble NA in its active tetrameric conformation, an imperative for the optimal induction of protective NA inhibitory antibodies. The impact of repetitive immunizations with TB-stabilized antigens on the immunogenicity of soluble TB-stabilized NA is unknown. We demonstrate that TB is immunogenic in mice. Interestingly, preexisting anti-TB antibodies enhance the anti-NA antibody response induced by immunization with TB-stabilized NA. This immune-enhancing effect was transferable by serum and operated independently of activating Fcγ receptors. We also demonstrate that priming with TB-stabilized NA antigens, enhances the NA inhibitory antibody responses against a heterosubtypic TB-stabilized NA. These findings have implications for the clinical development of oligomeric vaccine antigens that are stabilized by a heterologous oligomerizing domain.

## Introduction

The neuraminidase (NA) and hemagglutinin (HA) are the major surface antigens of influenza A and B virions. HA binds sialic acid, the receptor, on the surface of host cells whereas the catalytic activity of NA removes the receptor. The two proteins thus have complementary activities that have evolved into a functional balance that facilitates virus entry, budding, and potentially transmission^1–3^. Importantly, recent studies have highlighted that NA inhibitory (NAI) antibodies correlate with protection against seasonal and pandemic influenza^4–8^. Nevertheless, the presence of hemagglutination inhibition (HAI) antibodies in circulation remains the primary correlate of protection^9^ and currently licensed influenza vaccines aim primarily to induce seroprotective HAI titers. Unlike a natural infection, split inactivated influenza vaccines poorly induce NA-reactive antibodies^10,11^. In general, antigenic drift in NA of human influenza A viruses is considered to proceed at a slower pace compared to HA^12^. However, besides antigenic drift in NA as determined by the presence of NAI antibodies, drift may also result from non-NAI antibodies that recognize the lateral side of NA which can protect through Fcγ receptor-mediated mechanisms^13^. To improve the generally poor anti-NA response induced by current influenza vaccines, it has been suggested that supplementation of inactivated influenza vaccines with stabilized NA could potentially further improve protection^14,15^.

The production of stable, tetrameric NA is a critical requisite for its use as an immunogen that can induce NAI antibodies. Enzymatic activity of NA has been frequently assumed as an indication for its correct folding and oligomeric assembly. Influenza NA activity is only observed when the protein is assembled into a homo-tetramer^16,17^. It remains unclear why influenza NA is only active as a tetramer given that each protomer contains an active site. Possibly, contacts between NA protomers in the tetrameric assembly critically contribute to the stabilization of the catalytic site, as has been suggested by molecular dynamic simulations^18^. The tetrameric configuration of NA is promoted by the transmembrane domain^19^. Hence the production of stable, soluble tetrameric NA presents a challenge. This challenge has been addressed by fusing the NA head domain to a heterologous tetramerizing zipper such as the human vasodilator-stimulated phosphoprotein (VASP) domain, a tetramerizing domain derived from the yeast general control transcription factor GCN4, or at tetramerizing domain derived from tetrabrachion (TB)^20–22^. TB is a tetramerizing domain derived from the surface layer protein of the thermophile *Staphylothermus marinus* and forms a highly stable, right-handed parallel coiled-coil, which provides higher stability than VASP or GCN4 domains when fused to soluble NA^22–24^. We previously reported that the TB domain can be used to generate soluble tetrameric NA that is enzymatically active and can induce protective NAI responses^25^. Furthermore, crystal structure analysis showed that the fusion of TB to an N9 NA head domain faithfully mimics the structure of the corresponding native NA head domain^26^.

Although TB-stabilized NA, among other zippers, is being explored as an NAI antibody-inducing vaccine antigen, the immunogenicity of the TB and its impact on antigen immunogenicity upon repeated immunizations have not been evaluated. Evaluating the immunogenicity of TB is important since seasonal influenza vaccines are administered repeatedly during life. Here, we used TB fused to the domain III of human serum albumin (tetHSA) to induce preexisting anti-TB immune responses in mice. TB-primed mice were subsequently immunized with TB-NA antigen fusions derived from N1 or N2 NA. Data indicated that TB-specific serum IgG antibodies were readily induced by these fusion proteins. Interestingly, the presence of anti-TB IgG favored the NAI response elicited by subsequent immunization with TB-NA antigen. We also found that the enhanced NAI responses could be transferred by anti-TB IgG-containing immune serum yet was independent of activating Fcγ receptors.

## Results

### Tetrabrachion is immunogenic

**Fig. 1:**
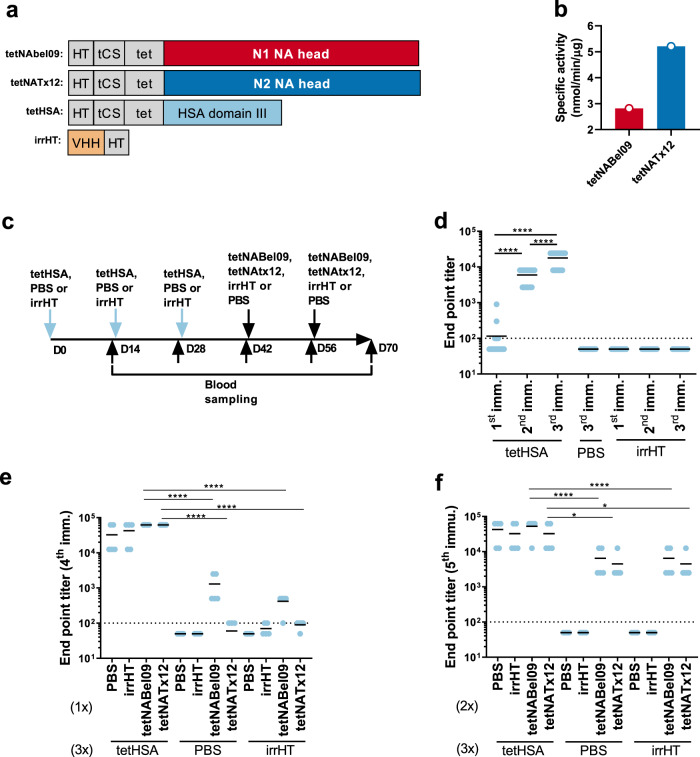
Tetrabrachion is immunogenic. **a** Schematic representation of the recombinant proteins that were used as immunogens. TB fusion proteins were constructed with the CD5 secretion signal, and the mature proteins contained a His-tag (HT), followed by a thrombin cleavage site (tCS), the TB coiled-coil domain (tet) followed by the head domain of Bel/09 NA (tetNABel09), the head domain of Tx12 (tetNATx12), or human serum albumin domain III (tetHSA). The irrelevant His-tagged (irrHT) protein is a single domain antibody (VHH) directed against Junin virus. **b** Specific activity of tetNA fusion proteins as determined in a MUNANA assay. **c** Mouse immunization scheme. Groups of 20 BALB/c mice received three immunizations at intervals of 2 weeks with either PBS, tetHSA, or irrHT (D0, D14, and D28). Six weeks after the first immunization groups of five mice were immunized with PBS, irrHT, tetNABel09, or tetNATx12, and this immunization was repeated 2 weeks later. Blood was collected 2 weeks after the prime, and prior to each subsequent immunization. **d**–**f** Serology. ELISA was performed using tetHSA captured in nickel-coated plates with sera isolated after the first, second, and third (**d**), the fourth (**e**), and fifth (**f**) immunization. Data were analyzed by one-way ANOVA, followed by Sidak’s multicomparison test, red horizontal bars represent means (*p < 0.05 and ****p < 0.0001). The dotted line in panels **d**–**f** represents the limit of detection, corresponding to the initial serum dilution used in the assay.

We generated three types of recombinant proteins to assess the potential immunogenicity of the non-NA components of the fusion proteins (Fig. 1a). First, we constructed recombinant TB-stabilized tetrameric N1 and N2 NA that was expressed in and purified from transiently transfected Chinese hamster ovary cells. These NA constructs comprised the full-length (50 amino acid residues) coiled-coil domain of TB genetically fused to the head domain of NA derived from H1N1pdm A/Belgium/145-MA/2009 (Bel09) or H3N2 A/Texas/50/2012 (Tx12). In addition, the TB-NA expression constructs carried a CD5 signal sequence and an N-terminal His-tag that was used for purification of the soluble tetrameric NA constructs. Secondly, domain III of human serum albumin protein fused to TB (tetHSA) was designed and produced. Finally, an irrelevant His-tagged protein (irrHT; a single domain antibody directed against Junin virus) was used as a control to evaluate the potential immunogenicity of the His-tag (Fig. 1a). The enzymatic activity of the resulting tetNABel09 and tetNATx12 proteins was confirmed using fluorogenic 4-methylumbelliferyl-*N*-acetylneuraminic acid (MUNANA) as a substrate, indicating that the proteins were correctly folded (Fig. 1b).

We hypothesized that the TB domain would be immunogenic and, since seasonal influenza vaccines are administered repeatedly throughout life, preexisting immune responses against TB might affect the immunogenicity of a subsequently administered TB-stabilized NA. To evaluate this, BALB/c mice were first immunized three times with tetHSA, irrHT, or PBS. Two weeks after the third immunization, the mice received two additional immunizations with tetNABel09_,_ tetNATx12, irrHT, or PBS as outlined in Fig. 1c. All immunizations were performed by intramuscular injection using AF03 as an adjuvant.

Two weeks after the first tetHSA immunization, the majority of the mice had undetectable anti-tetHSA IgGs. However, after a second dose, all the tetHSA immunized mice had seroconverted and anti-tetHSA titers were boosted further after the third dose of this immunogen. As expected, anti-tetHSA serum IgG responses were undetectable in animals that received PBS (Fig. 1d). Serum from mice that had been immunized three times with irrHT did not show detectable antibodies against tetHSA, suggesting the absence of anti-His-tag antibodies (Fig. 1d). After the first immunization with tetNABel09 or tetNATx12, anti-tetHSA serum IgG titers increased further in mice that had been previously immunized with tetHSA but not anymore after the second immunization with TB-NA (fifth immunization in total) in those mice. Interestingly, anti-tetHSA IgG titers were also induced by immunization of PBS- or IrrHT-primed mice with tetNABel09 and tetNATx12 (Fig. 1e, f). These results suggest that the TB domain and/or the His-tag in TB-NA is immunogenic. However, immunization with tetNAs did not induce detectable IgG titers against the irrelevant His-tagged protein indicating that the His-tag in tetNA is poorly immunogenic in mice (Supplementary Fig. 1a, b).

### Preexisting anti-TB immune responses increase NAI responses induced by tetNA immunization

**Fig. 2:**
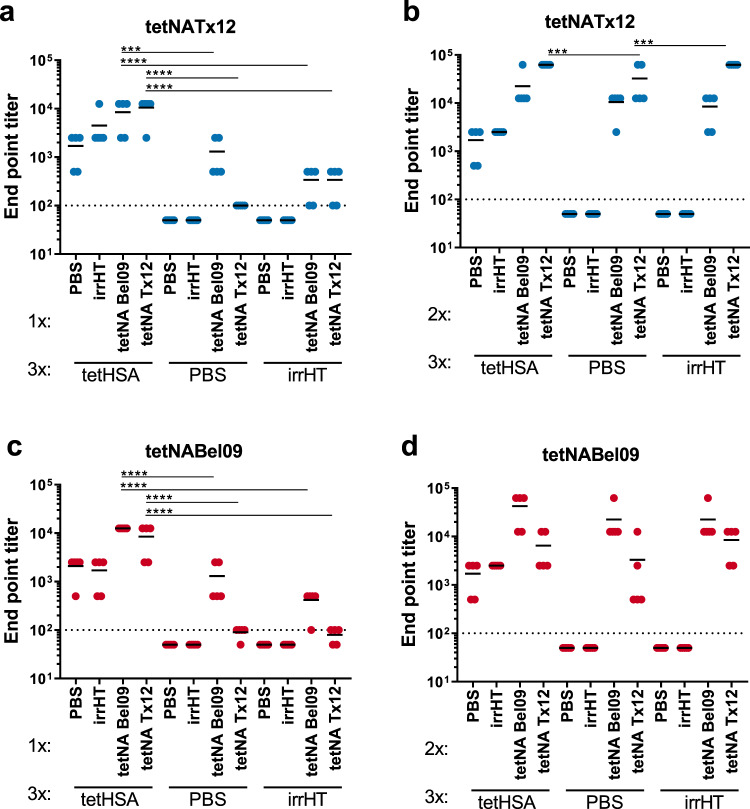
TetHSA priming is associated with an increased anti-NA response. TetNA-specific serum IgG titers were determined by ELISA with tetNATx12 (**a**, **b**) or tetNABel09 (**c**, **d**) captured in wells of Ni NTA coated plates. **a** Anti-tetNATx12 titers after the fourth and **b** fifth immunization. Anti-tetNABel09 titers after the fourth (**c**), and fifth (**d**) immunization. Data were analyzed by one-way ANOVA, followed by Sidak’s comparison test, bars represent the mean (***p < 0.001, ****p < 0,0001). The dotted line represents the limit of detection, corresponding to the initial serum dilution used in the assay.

We assessed the possible impact of preexisting anti-TB responses on subsequent immunization with TB-stabilized soluble N1 or N2 NA. Quantification of serum anti-tetNA IgGs by capture ELISA showed that mice that had been primed by immunization with tetHSA had higher anti-tetNA titers after the first immunization with tetNABel09 or tetNATx12 compared to the mice that had been primed with PBS or the irrelevant His-tagged protein (Fig. 2a, c). This difference was less apparent after the second dose of tetNA (Fig. 2b, d).

**Fig. 3:**
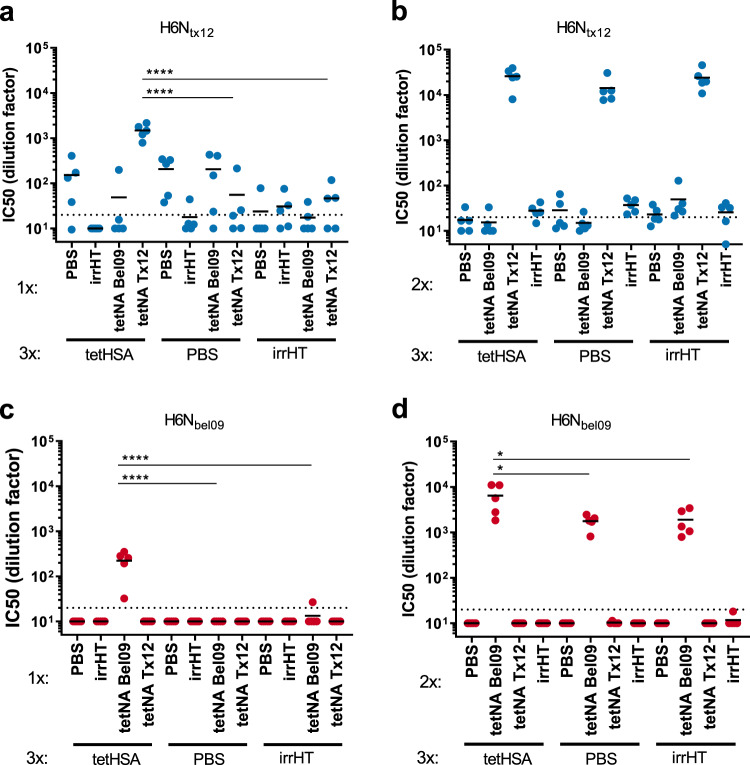
Neuraminidase inhibition titers are increased in tetHSA-primed mice. NAI was determined in sera collected after the fourth (**a**, **c**) and fifth immunization (**b**, **d**). The reassortant viruses H6N_tx12_ (**a**, **b**) and H6N_bel09_ (**c**, **d**) were used as a source of NA in the ELLA. Data shows the IC_50_ values as determined by nonlinear regression analysis and plotted as the values of 1:x dilution resulting in 50% NA inhibition. Data were analyzed by one-way ANOVA and Sidak’s comparison test, bars represent means (*p = 0.03, ****p < 0.0001). The dotted line represents the limit of detection, corresponding to the initial serum dilution used in the assay.

To assess whether the increased anti-TB-NA IgG titer that was observed in tetHSA-primed mice that had been boosted with TB-NA was not merely due to increased anti-TB responses, NAI titers in the different sera were determined. NAI assays were performed with sera collected after the fourth and fifth immunization using H6N1 and H6N2 reassortant viruses that carried homologous NA as targets in an enzyme-linked lectin assay (ELLA). After the first immunization with tetNA, the calculated NAI IC_50_ values for H6N_bel09_ and H6N_tx12_ were significantly higher in mice that had been pre-immunized with tetHSA compared to mice that had been primed with PBS or the irrelevant His-tagged protein (Fig. 3a, c). The increased serum NAI in tetHSA-primed mice was still observed after a second immunization with tetNABel01 but not with tetNATx12 (Fig. 3d, b, respectively). Interestingly, priming of mice with tetNABel09 (2x) followed by two booster immunizations with tetNATx12 or vice versa, also resulted in a significantly higher NAI titer compared to PBS-primed mice (Supplementary Fig. 2). These data suggest that preexisting antibodies directed against TB contribute to an increased immune response induced by a subsequent immunization with soluble tetNA.

### The immune-enhancing effect induced by tetHSA is transferable by serum

**Fig. 4:**
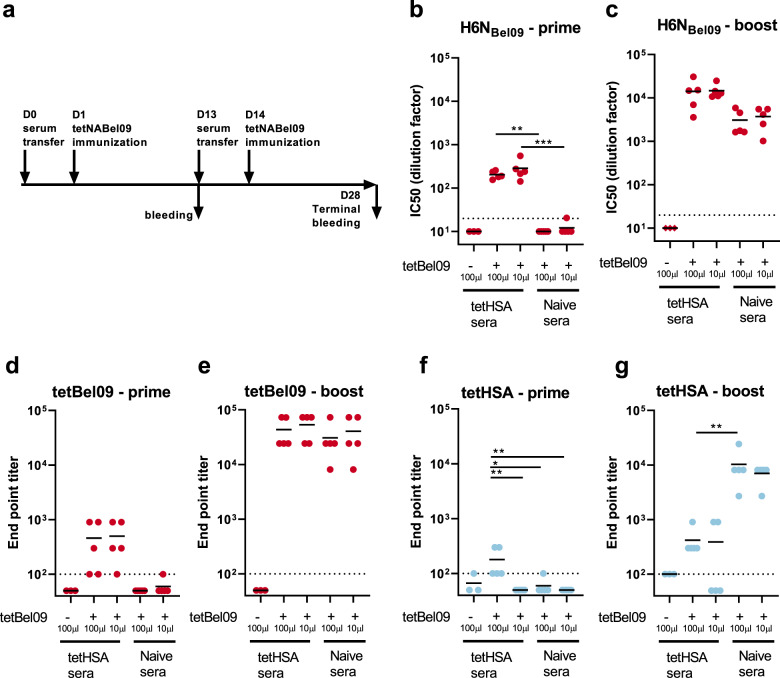
Passive transfer of tetHSA immune serum enhances NAI antibodies. **a** Immunization scheme. BALB/c mice received 100 or 10 µl of anti-tetHSA or naïve serum intraperitoneally on days 0 and 13 and were immunized the following day with tetNABel09 (+) or PBS control (−). Blood was sampled on days 13 and 28. **b**, **c** NAI against H6N_bel09_ determined in sera collected 2 weeks after the first (**b**) and second immunization (**c**). Data show the IC_50_ values as determined by nonlinear regression analysis and plotted as the values of 1:x dilution resulting in 50% NA inhibition. **d**, **e** Anti-tetNABel09 IgG titers determined by ELISA after the first (**d**) and second (**e**) immunization. **f**, **g** Anti-tetHSA responses after the first (**f**) and second (**g**) immunization. Data were analyzed by one-way ANOVA, followed by Sidak’s comparison test, bars represents means (*p < 0.05, **p < 0.01, and ***p < 0.001). The dotted line in panels **b**–**g** represents the limit of detection, corresponding to the initial serum dilution used in the assay.

To test to which extent the increased immunogenicity of tetNABel09 in tetHSA-primed mice is dependent on the humoral response, serum obtained from tetHSA- or control-immunized mice was transferred to naïve animals one day prior to immunization with tetNABel09. This procedure was repeated two weeks later to recapitulate the active tetNA prime-boost immunization regimen described above (Fig. 4a). IgG1 was the predominant tetHSA-reactive IgG subclass in the transferred sera, followed by IgG2a and IgG3 whereas a low titer of IgM and no IgG2b was detectable (Supplementary Fig. 3). Mice that were pretreated with 10 or 100 µl of tetHSA serum given intraperitoneally responded with a significantly higher NAI IC_50_ titer after the first tetNA immunization when compared to mice that had received naive serum (Fig. 4b). A similar trend was observed following the second serum treatment followed by immunization, although a statistically significant difference was not obtained (Fig. 4c).

The tetHSA serum transfer followed by the first immunization with tetNABel09 induced higher, although not statistically significantly different, IgG titers against immobilized tetNABel09, compared to the response in control serum recipients (Fig. 4d). On day 28, 2 weeks after the second immunization with tetNABel09, the anti-tetNABel09 serum IgG titers were comparable in all four groups of mice (Fig. 4e). Anti-tetHSA titers were higher in the mice that received 100 µl of tetHSA serum 2 weeks after the first immunization with tetNABel09 (Fig. 4f). However, after the second tetNABel09 immunization the anti-tetHSA IgG titers were significantly lower in mice that had received anti-tetHSA serum prior to tetNABel09 immunization when compared to mice that had received sera from naïve mice (Fig. 4g). These data indicate that serum with anti-TB antibodies can promote NAI responses induced by tetNA whereas such antibodies, at the same time, are associated with a reduced anti-TB antibody response induced by a TB-carrying antigen.

### Increased tetNA immunogenicity by preexisting anti-TB antibodies does not rely on activating Fcγ receptors

**Fig. 5:**
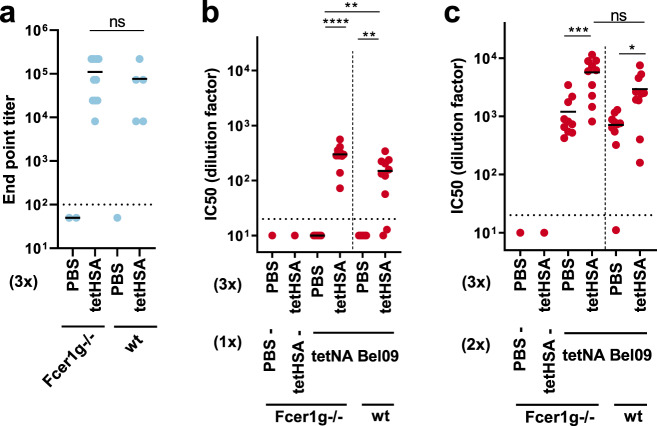
NAI responses are increased in TB-primed wild-type and *Fcerg1*^−/−^ mice. **a** ELISA titers against tetHSA in sera isolated after three immunizations with PBS or tetHSA in wt and *Fcer1g*^−/−^ mice. **b**, **c** NAI determined against H6N_bel09_ in sera collected after the fourth (**b**) and fifth immunization (**c**). Data show the IC_50_ values as determined by nonlinear regression analysis and plotted as the values of 1:x dilution resulting in 50% NA inhibition. Data were analyzed by one-way ANOVA and Sidak’s comparison test, bars represent means (*p = 0.02, **p < 0.01, ***p = 0.0002, ****p < 0.0001). The data points are compiled from two independently performed experiments. The dotted line represents the limit of detection, corresponding to the initial serum dilution used in the assay.

Antibodies can form immune complexes that promote adaptive immune responses against the complexed antigen by the interaction of the Fc domain of these antibodies with activating Fcγ receptors such as Fcγ RI^27^. To evaluate the possible role of activating Fcγ receptors in the enhanced NAI response in the presence of preexisting anti-TB antibodies, *Fcer1g*^−/−^ and wild-type (wt) BALB/c mice were immunized three times with tetHSA, followed by two immunizations with tetNABel09. In mice, *Fcer1g*, codes for the common γ-chain of the activating Fcγ receptors I, III, and IV^28^. There was no difference in the anti-tetHSA serum IgG response between wild-type and *Fcer1g*^−/−^ mice (Fig. 5a). As before, tetHSA-primed wild-type mice displayed significantly higher serum NAI levels after the first immunization with tetNABel09 compared to the PBS-primed mice (Fig. 5b). Interestingly, this difference in NAI levels between PBS- and tetHSA-primed animals was even more pronounced in the *Fcer1g*^−/−^ mice (Fig. 5b). Furthermore, this difference still reached statistical significance after a boost immunization with tetNABel09 of *Fcer1g*^−/−^ mice and wt mice (Fig. 5c). We conclude that activating FcγR signaling in mice is not required for the immune-stimulating effect of preexisting anti-tetHSA immune responses against subsequent immunization with tetNA with a shared TB-based tetramerizing domain.

## Discussion

The fusion of NA to the coiled-coil domain of TB was shown to stabilize the tetrameric head domain of NA, while allowing correct folding, and enabled the large-scale production and purification of homogenous, batch-to-batch consistent NA antigen. Here we showed that TB fused to the control protein HSA, NA from group 1 (tetNABel09) or group 2 (tetNATx12), is immunogenic in mice. Interestingly, preexisting anti-TB antibodies were associated with the induction of increased IgG and NAI titers after a subsequent immunization with tetNA. Such effect was observed when mice were primed with control tetHSA protein and also in a heterologous tetN1/N2 prime-boost regimen. Similar to the active immunization, the transfer of serum from mice immunized with tetHSA also resulted in increased IgG and NAI titers after subsequent tetNA immunization. This observation indicates that the enhanced immune response is mediated by anti-tetHSA antibodies but does not exclude a possible contribution of a helper T cell response directed against one or more T helper epitopes in the tetramerizing domain of tetrabrachion in the active immunization setting. Further, passive transfer of serum with anti-tetHSA IgG was associated with reduced anti-TB IgG induction after immunization with tetNABel09.

The modulation of the humoral response by antibodies relies on different mechanisms that can either lead to suppression or enhancement of this response^29^. For example, a reduction of antibody induction has been previously described in the context of the anti-HA response, which was attributed to antigenic masking by the preexisting anti-HA antibodies^30–32^. Here, the small size of the TB domain (6 kDa) relative to NA (44 kDa) may facilitate antigenic masking by antibodies and the consequent suppression of the anti-TB response, which we observed in the passive transfer experiment (Fig. 4g)^33^. The reduced response was TB-specific, suggesting that anti-TB antibodies do not sterically interfere with the recognition of epitopes on the NA head domain. A reduced anti-TB response was not observed in the active immunization setting upon subsequent immunization with tetNA, suggesting that antigenic masking is not sufficient to modulate anti-TB IgGs. We speculate that this may be due to our experimental design that made use of three immunizations to saturate the humoral anti-TB response. Alternatively, T helper cells directed against the TB domain induced by active immunization may have contributed to antigen presentation in trans.

In addition to the suppression of the anti-TB response in the serum transfer experiment, another important observation in our study is the enhancement of antibody responses against the TB-fused NA, which was observed after either active or passive immunization. Different Ab subclasses have been described as being able to mediate enhanced immune responses. The mechanism of enhancement may involve the recognition of antibody-antigen immune complexes by Fcγ receptors, complement receptors, or FcRn^27,33,34^. Previous studies using monoclonal antibodies complexed with antigens and knock-out mice have shown that Fcγ receptors are required for IgG1 and IgG2a-mediated enhancement of the immune response against the hapten trinitrophenyl^27^. IgG3 and IgGM have been described to promote the antibody response through a complement-dependent mechanism^35^. Here we show that the level of enhancement achieved after active tetHSA immunization in *Fcer1g*^−/−^ mice, which lack functional FcγRI, FcγRIII, FcγRIV, and FcεRI, is even slightly higher than the immune-stimulating effect observed in the wild-type mice. This result indicates that FcγRI, FcγRIII, FcγRIV, and FcεRI, have no significant role or their contribution can be compensated by other mechanisms (e.g., complement- or FcRn-dependent). The complexity of polyclonal sera imposes limitations in the dissection of these mechanisms in our model. We hypothesize that preexisting anti-TB IgG3 can form immune complexes that favor the transport and availability of antigen in the lymphoid organs through follicular dendritic cells^36^. Such IgG3-mediated immune complexes could be formed with complement, and it has been reported that lack of C1q or C3 impairs the ability of IgG3 to enhance antibody responses in mice^37^.

In summary, our results indicate no deleterious, but rather an immune-enhancing effect of repeated TB immunization for the generation of immune responses against TB-stabilized NA. Whether such an enhancing effect of repeat immunization of TB-stabilized tetrameric NA would also be apparent in a clinical setting remains to be determined.

## Methods

### Design and production of recombinant proteins

The coding information for TB-NA and TB-HSA fusion proteins was cloned under the transcriptional control of the CMV promoter in the pCDNA3.4 plasmid with a CD5 secretion signal, an amino-terminal His-tag, and the thrombin cleavage signal followed by tetrabrachion-NA or tetrabrachion-HSA domain III. TB-NA and TB-HSA were expressed in a mammalian cell culture system as previously described with modifications^38^.

The irrelevant His-tagged protein used is a single domain antibody directed against the Junín virus, which was produced in *P. pastoris* as described previously^39^. Briefly, 48 h after methanol induction, cells were pelleted and the growth medium was subjected to ammonium sulfate precipitation (80% saturation). The insoluble fraction was pelleted by centrifugation at 20,000×*g* and the pellet was solubilized in 10 ml of HisTrap binding buffer (20 mM sodium phosphate, 0.5 M NaCl, 20 mM imidazole, pH 7.4) before purification on a 1 ml HisTrap HP column (GE Healthcare).

### Recombinant NA activity measurement

The enzymatic activity of tetNABel09 and tetNATx12 was determined with the fluorogenic small substrate 4-methylumbelliferyl)-α-d-*N*-acetylneuraminic acid (MUNANA) as follows. Ten microliters of 1 mM of 4-MUNANA (Sigma cat # M8639) in 200 mM sodium acetate buffer (pH 6.5) containing 2 mM CaCl_2_ and 1% butanol was incubated with 40 µl of PBS solution containing 30, 15, 7.5, or 3.75 ng of tetNA. Conversion to 4-methylumbelliferone (4-MU) was monitored every 2 min for 1 h using BMG Fluostar OPTIMA reader (excitation at 365 nm and emission determined at 450 nm). A standard curve with 800 to 25 pmoles/well of 4-methylumbelliferyl (Sigma cat # M1508) was used to extrapolate the molar conversion of MUNANA per microgram of purified recombinant tetNABel09 and tetNATx12.

### Mouse immunization experiments

All animal experiments were conducted according to the Belgian legislation (Belgian Law 14/08/1986 and Belgium Royal Decree 06/04/2010) and European legislation on protection of animals used for scientific purposes (EU directives 2010/63/EU and 86/609/EEC). Experimental protocols were all approved by the Ethics Committee of the Vlaams Instituut voor Biotechnologie (VIB), Ghent University, Faculty of Science (permit numbers EC016-059 and EC2019-035). Female BALB/c mice, aged 6–7 weeks, were purchased from Charles River (France) and *Fcer1g*^−/−^ mice on the BALB/c background were from Taconic Biosciences. The animals were housed in a specified pathogen-free animal house with food and water ad libitum. Animals were immunized intramuscularly in the right quadriceps. Immunization was performed with 50 µl containing 1 µg of recombinant protein (tetHSA, tetNABel09, tetNATx12, or irrHT). All immunizations were adjuvanted with a 1:1 volume of AF03 (25 μl of antigen in PBS + 25 μl of AF03 per dose)^40^. In serum transfer experiments animals received 100 or 10 µl of PBS- or tetHSA immune serum, pooled from individual mice that had been immunized three times with tetHSA or PBS in the presence of AF03 adjuvant. Serum (100 or 10 µl adjusted with 90 µl PBS to 100 µl total volume) was injected intraperitoneally 1 day prior to tetNA immunization.

Two weeks after each immunization, 75–150 µl of blood was collected by puncturing the lateral tail vein with a 23 G needle. The obtained blood samples were incubated for 30 min at 37 °C to allow clotting, which was followed by centrifugation at 10,500 × *g* for 5 min. The supernatant (the serum) was recovered and submitted to a second centrifugation at 10,500×*g* for 5 min. Cleared sera were stored at −20 °C before use in serological assays. Before terminal retro-orbital bleeding, animals were first humanely sacrificed with an anesthetic overdose of Nembutal (6 mg per mouse).

### ELISA

Anti-tetNA and anti-tetHSA IgG levels in mouse sera were determined by capture ELISA using tetNABel09, tetNATx12 or tetHSA in Pierce^TM^ nickel-coated plates (cat. # 15442). To determine anti-irrHT serum IgG titers, irrHT was directly coated on Maxisorp^TM^ plates (Thermofisher cat. # 44-2404-21). Recombinant proteins were diluted in DPBS (Life technologies cat. # 14040-182) (tetNA and irrHT was diluted to 0.5 µg/ml and tetHSA to 0.29 µg/ml). Then, 50 µl of the coating antigen solutions was added to each well and the plate was subsequently incubated at room temperature for 1 h on a shaking platform. The wells of the plates were then washed three times with PBS-T (Sigma cat. # P3563-10PAK) and blocked for 1 h with 1% BSA in DPBS. After blocking, the wells of the plates were washed once with PBS-T and incubated with a threefold serial dilution, starting at a 1/100 dilution, of serum in DPBS, 0.5% BSA, 0.05% tween20 for 2 h at room temperature on a shaking platform. The wells of the plates were then washed five times with PBS-T and incubated with a 1:4000 dilution of anti-mouse IgG-HRP, (GE healthcare cat. # NA931-1ml), anti-mouse IgG3-HRP (SouthernBiotech cat. # 1100-08), anti-mouse IgG2a-HRP (SouthernBiotech cat. #1080-05), anti-mouse IgG2b-HRP (SouthernBiotech cat. # 1090-05), anti-mouse IgG1-HRP (SouthernBiotech cat.#1070-01), or 1:5000 dilution of anti-mouse IgM-HRP (Bio-RAD STAR86), in PBS, 0.5% BSA, 0.05% Tween20. The 3,3′,5,5′-tetramethylbenzidineTMB substrate (TMB) (BD cat. # 555214) was added after three washes with PBS-T and the reaction was stopped after 5 min by addition of 50 µl of 1 M H_2_SO_4_. The optical density (OD) in each well was determined at 450 nm and, as a reference, 655 nm using an iMark™ Microplate Absorbance Reader (Bio-rad). The end point titer was determined for each serum sample by scoring the dilution that resulted in an OD that was equal to or higher than two times the background OD obtained from the pre-immune control sera dilution series.

### Enzyme-linked lectin assay to determine neuraminidase inhibition titers

Fetuin (Sigma cat. # F3385) was diluted into coating buffer (KPL cat. # 50-84-01) to a concentration of 25 µg/ml and 50 µl was added to the wells of Nunc MaxiSorp™ plates (Thermofisher cat. # 44-2404-21), which were incubated overnight at 4 °C. The coated plates were then washed three times with PBS-T (Sigma cat. # P3563-10PAK) and incubated overnight with 60 μl of a 1/327 dilution of H6N1_A/Belgium/145/2009_ or 1/220 dilution of H6N2_A/Texas/50/2012_ and 60 µl of 2-fold serial dilution of serum, starting at a 1/20 dilution, in sample buffer (1X MES VWR cat. # AAJ61979-AP: 20 mM CaCl_2_, 1% BSA, 0.5% Tween20). These dilutions of the H6N_x_ viruses correspond to the 70% maximum activity of NA from the respective viruses as determined in the ELLA assay. Fetuin-coated plates were then washed three times with PBS-T and incubated for 1 h with a solution of PNA-HRP (cat. # L6135-1MG, Sigma) at 5 μg/ml in conjugate diluent (MES pH 6.5, 20 mM CaCl2, 1% BSA). The plates were washed three times with PBS-T, TMB substrate was added and then the plates were incubated for 5 min before the reaction was stopped by the addition of 50 µl of 1 M H_2_SO_4_. The optical density was measured at 450 nm and as a reference 655 nm in an iMark™ Microplate Absorbance Reader (Bio-rad). Half maximum inhibitory concentrations (IC_50_) values were determined by nonlinear regression analysis (GraphPad Prism software).

### Statistical analysis

Results are presented as individual data points with the mean indicated. Quantitative variables were tested using one-way ANOVA and where significant differences were found, post hoc analysis using the Sidak test was performed. GraphPad Prism version 8.3.0 was used for analysis and statistical significance was considered when *p* < 0.05.

### Reporting Summary

Further information on research design is available in the Nature Research Reporting Summary linked to this article.

## Supplementary information

Supplemental information revised

REPORTING SUMMARY

## Data Availability

The data that support the findings of this study are available from the corresponding authors upon request.
