# Supplementary material for: Pre-existing antibodies directed against a tetramerizing domain enhance the immune response against artificially stabilized soluble tetrameric influenza neuraminidase
Source: NPJ Vaccines. 2022 Jan 27;7:11. doi: 10.1038/s41541-022-00435-7 (PMC8795415; doi:10.1038/s41541-022-00435-7)
Supplement: Supplementary file 1 — Supplemental information revised [file 41541_2022_435_MOESM1_ESM.pdf]

Supplementary material for:

Pre-existing antibodies directed against a tetramerizing domain enhance the immune response against artificially stabilized soluble tetrameric influenza neuraminidase

João Paulo Portela Catani[1,2], Emma R. Job[1,2,&], Tine Ysenbaert[1,2], Anouk Smet[1,2], Satyajit Ray[3], Lauren LaRue[3], Svetlana Stegalkina[3], Mario Barro[3], Thorsten U. Vogel[3,*], Xavier Saelens[1,2,*]

[1]VIB-UGent Center for Medical Biotechnology, VIB, B-9052 Ghent, Belgium

[2]Department of Biochemistry and Microbiology, Ghent University, B-9000 Ghent, Belgium

[3]Sanofi Pasteur, Research North America, Cambridge, Massachusetts, USA

[&]Current affiliation: Janssen Infectious Diseases and Vaccines, Janssen Research and Discovery, Beerse, Belgium

[*]Correspondence: Xavier Saelens (Xavier.Saelens@vib-ugent.be) or Thorsten U. Vogel (Thorsten.Vogel@sanofi.com)

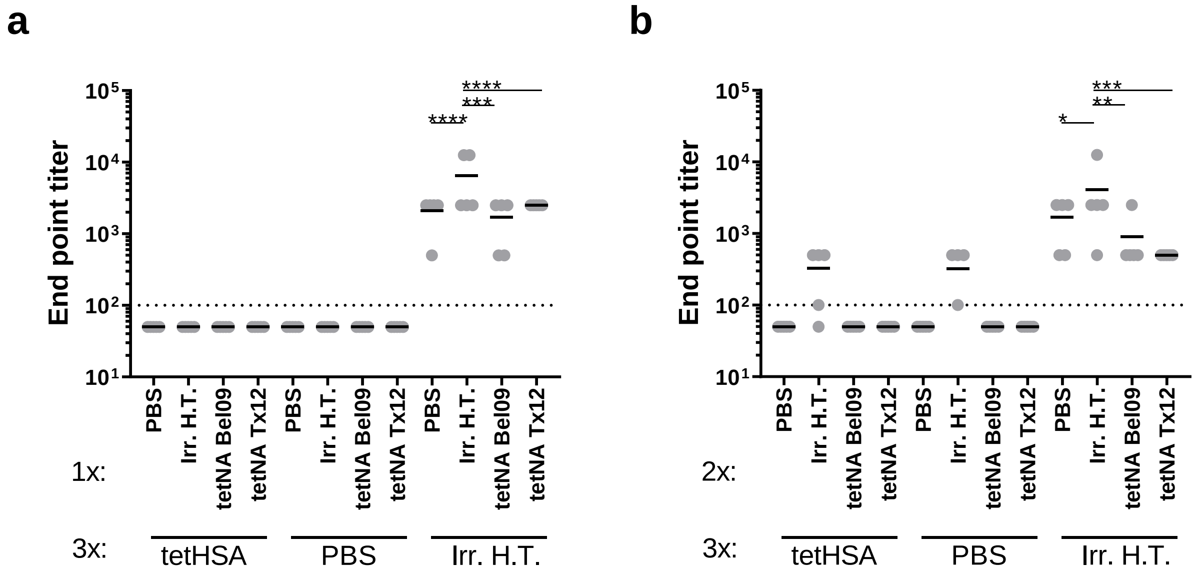

Supplementary Figure 1. Serum IgG titers against irrelevant His-tagged protein. Mice were immunized as outlined in Figure 1 and ELISA was performed using the irrHis-tagged protein coated in wells of Maxisorp™ plates. Sera obtained after the 4th (a) and 5th (b) immunization were tested. Data were analyzed by one-way ANOVA, followed by Sidak's multicomparison test, horizontal bars represent means (*P<0.05, **P<0.01, ***P<0.001, ****P<0.0001). The dotted line represents the limit of detection, corresponding to the initial serum dilution used in the assay.

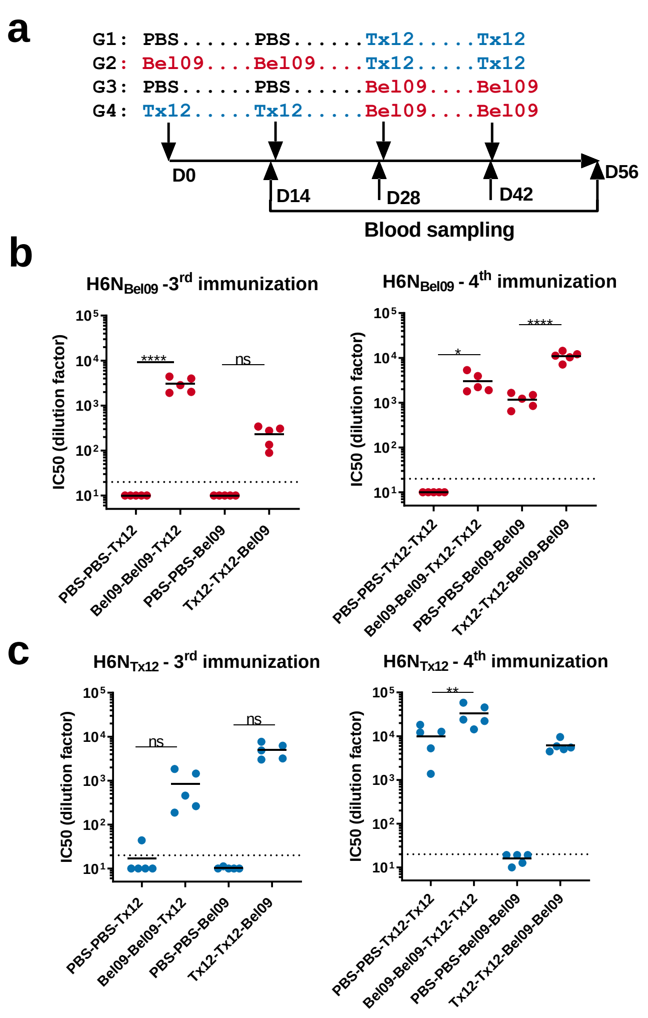

Supplementary Figure 2. Heterologous prime-boost with N1 and N2 tetNA promotes NA inhibition responses. (a) Immunization scheme. Four groups of 5 BALB/c mice were immunized 4 times with PBS, tetNABel09 (Bel09), or tetNATx12 (Tx12) as indicated. (b, c) NA inhibition titers in serum determined in ELLA using H6N1$_{Bel09}$ (b) or H6N2$_{Tex12}$ (c). ELLA titers in sera obtained after the 3rd (left) and 4th (right) immunization were determined. Data shows the IC_{50} values as determined by non-linear regression analysis and plotted as the values of 1:x dilution resulting in 50% NA inhibition. Horizontal bars represent means and statistical significance was determined using one-way ANOVA, followed by Sidak's multicomparison test (*P<0.05, **P<0.01, ****P<0.0001). The dotted line in panels b and c represents the limit of detection, corresponding to the initial serum dilution used in the assay.

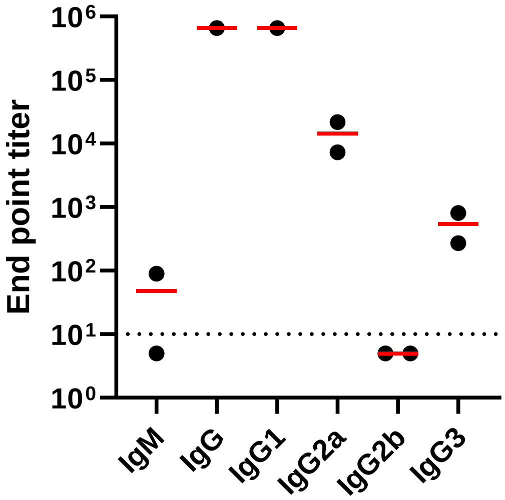

Supplementary Figure 3. Immunoglobulin subclasses induced by tetHSA immunization. IgM and IgG subclasses directed against tetHSA were quantified by ELISA in pooled sera from BALB/c mice that were immunized 3 times with tetHSA. The dotted line represents the limit of detection, corresponding to the initial serum dilution used in the assay.
