# Supplementary material for: Pre-existing antibodies directed against a tetramerizing domain enhance the immune response against artificially stabilized soluble tetrameric influenza neuraminidase
Source: NPJ Vaccines. 2022 Jan 27;7:11. doi: 10.1038/s41541-022-00435-7 (PMC8795415; doi:10.1038/s41541-022-00435-7)
Supplement: Supplementary file 2 — REPORTING SUMMARY [file 41541_2022_435_MOESM2_ESM.pdf]

nature portfolio

Corresponding author(s): Professor Xavier Saelens

Last updated by author(s): 25.11.2021

Reporting Summary

Nature Portfolio wishes to improve the reproducibility of the work that we publish. This form provides structure for consistency and transparency in reporting. For further information on Nature Portfolio policies, see our Editorial Policies and the Editorial Policy Checklist.

Statistics

For all statistical analyses, confirm that the following items are present in the figure legend, table legend, main text, or Methods section.

n/a	Confirmed	
☐	☒	The exact sample size (n) for each experimental group/condition, given as a discrete number and unit of measurement
☒	☐	A statement on whether measurements were taken from distinct samples or whether the same sample was measured repeatedly
☐	☒	The statistical test(s) used AND whether they are one- or two-sided<br>*Only common tests should be described solely by name; describe more complex techniques in the Methods section.*
☒	☐	A description of all covariates tested
☒	☐	A description of any assumptions or corrections, such as tests of normality and adjustment for multiple comparisons
☒	☐	A full description of the statistical parameters including central tendency (e.g. means) or other basic estimates (e.g. regression coefficient) AND variation (e.g. standard deviation) or associated estimates of uncertainty (e.g. confidence intervals)
☐	☒	For null hypothesis testing, the test statistic (e.g. F, t, r) with confidence intervals, effect sizes, degrees of freedom and P value noted<br>*Give P values as exact values whenever suitable.*
☒	☐	For Bayesian analysis, information on the choice of priors and Markov chain Monte Carlo settings
☒	☐	For hierarchical and complex designs, identification of the appropriate level for tests and full reporting of outcomes
☒	☐	Estimates of effect sizes (e.g. Cohen's d, Pearson's r), indicating how they were calculated

Our web collection on statistics for biologists contains articles on many of the points above.

Software and code

Policy information about availability of computer code

Data collection	*Provide a description of all commercial, open source and custom code used to collect the data in this study, specifying the version used OR state that no software was used.*
Data analysis	*Provide a description of all commercial, open source and custom code used to analyse the data in this study, specifying the version used OR state that no software was used.*

For manuscripts utilizing custom algorithms or software that are central to the research but not yet described in published literature, software must be made available to editors and reviewers. We strongly encourage code deposition in a community repository (e.g. GitHub). See the Nature Portfolio guidelines for submitting code & software for further information.

Data

Policy information about availability of data

All manuscripts must include a data availability statement. This statement should provide the following information, where applicable:

- Accession codes, unique identifiers, or web links for publicly available datasets
- A description of any restrictions on data availability
- For clinical datasets or third party data, please ensure that the statement adheres to our policy

The data that support the findings of this study are available from the corresponding authors upon request.

Field-specific reporting

Please select the one below that is the best fit for your research. If you are not sure, read the appropriate sections before making your selection.

☒ Life sciences ☐ Behavioural & social sciences ☐ Ecological, evolutionary & environmental sciences

For a reference copy of the document with all sections, see nature.com/documents/nr-reporting-summary-flat.pdf

Life sciences study design

All studies must disclose on these points even when the disclosure is negative.

Sample size	Sample size chosen was based on previous experience of experiments performed in the past concerning the mouse model of influenza. We aim to obtain statistically meaningful data between groups whilst taking the RRR ethical principals into consideration.
Data exclusions	No data were excluded from the analysis.
Replication	The passive serum transfer experiment, the results of which are described in Figure 5, was repeated once and the data were pooled to generate the graphs shown in Figure 5. Active immunization experiments were not repeated.
Randomization	Mice received from supplier were randomly assorted into cages and subsequently assigned treatment groups.
Blinding	Studies were not blinded. The majority of the practical work was performed by a small team making blinding difficult as members of the team were involved in every aspect from setting up the experiments to follow-up.

Reporting for specific materials, systems and methods

We require information from authors about some types of materials, experimental systems and methods used in many studies. Here, indicate whether each material, system or method listed is relevant to your study. If you are not sure if a list item applies to your research, read the appropriate section before selecting a response.

Materials & experimental systems

n/a	Involved in the study	
☐	☒	Antibodies
☐	☒	Eukaryotic cell lines
☒	☐	Palaeontology and archaeology
☐	☒	Animals and other organisms
☒	☐	Human research participants
☒	☐	Clinical data
☒	☐	Dual use research of concern

Methods

n/a	Involved in the study	
☒	☐	ChIP-seq
☒	☐	Flow cytometry
☒	☐	MRI-based neuroimaging

Antibodies

Antibodies used

anti-mouse IgG-HRP, (GE healthcare cat. # NA931-1ml), anti-mouse IgG3-HRP (SouthernBiotech cat. # 1100-08), anti-mouse IgG2a-HRP (SouthernBiotech cat. #1080-05), anti-mouse IgG2b-HRP (SouthernBiotech cat. # 1090-05), anti-mouse IgG1-HRP (SouthernBiotech cat. # 1070-01), anti-mouse IgM-HRP (Bio-RAD STAR86).

Validation

For the validation of these antibodies, we refer to the statements on the following websites of the respective providers:
anti-mouse IgG-HRP, (GE healthcare cat. # NA931-1ml):
https://d3.cytivalifesciences.com/prod/COFA/NA931-1ml_17212127.pdf
anti-mouse IgG3-HRP (SouthernBiotech cat. # 1100-05):
https://www.southernbiotech.com/?catno=1100-05&type=Polyclonal#&panel1-2&panel2-1
anti-mouse IgG2a-HRP (SouthernBiotech cat. #1080-05):
https://www.southernbiotech.com/?catno=1080-05&type=Polyclonal#&panel1-1&panel2-1
anti-mouse IgG2b-HRP (SouthernBiotech cat. # 1090-05):
https://www.southernbiotech.com/?catno=1090-05&type=Polyclonal#&panel1-1&panel2-1
anti-mouse IgG1-HRP (SouthernBiotech cat.#1070-01):
https://www.southernbiotech.com/?catno=1070-01&type=Polyclonal#&panel1-1&panel2-1
anti-mouse IgM-HRP (Bio-RAD STAR86):
https://www.bio-rad-antibodies.com//polyclonal/mouse-igm-antibody-star86.html?f=purified&_gl=1*11zuqoc*_gcl_aw*R0NMLjE2MzUyNjIzODAuQ2p3S0NBand6dDZMQmhCZUVpd0FiUEdPZ1lfaWJ4eVo2OHlLdlR0Q21oYTQ2d0NVTW9sU0dPaDZfQzdhcmloMENkWVVCWi1qZkZxb3d4b0NBVEVRQXZEX0J3RQ..&_ga=2.179612386.712794461.1635258829-251477161.1635258827&_gac=1.28854862.1635262380.CjwKCAjwzt6LBhBeEiwAbPGOgY_ibxyZ68yKvTtCmha46wCUMolSGOh6_C7arih0CdYUBZ-jfFqowxoCATEQAvD_BwE

Eukaryotic cell lines

Policy information about cell lines

Cell line source(s)	ATCC
Authentication	None of the cell lines were authenticated
Mycoplasma contamination	MDCK tested negative for mycoplasma contamination
Commonly misidentified lines (See ICLAC register)	N/A

Animals and other organisms

Policy information about studies involving animals; ARRIVE guidelines recommended for reporting animal research

Laboratory animals	Mus musculus, BALB/cAnNCrl, female, 6 weeks old
Wild animals	Study did not involve wild animals
Field-collected samples	Study did not involve field-collected samples
Ethics oversight	The ethics committee of the Vlaams Instituut voor Biotechnologie (VIB), Ghent University, Faculty of Science approved the mouse experiments.

Note that full information on the approval of the study protocol must also be provided in the manuscript.
